# Pandemic (H1N1) 2009 Risk for Nurses after Trivalent Vaccination

**DOI:** 10.3201/eid1604.091588

**Published:** 2010-04

**Authors:** Mark Loeb, David J.D. Earn, Marek Smieja, Richard Webby

**Affiliations:** McMaster University, Hamilton, Ontario, Canada (M. Loeb, D.J.D. Earn, M. Smieja); St. Jude Children’s Research Hospital, Memphis, Tennessee, USA (R. Webby)

**Keywords:** Influenza, H1N1, vaccine, viruses, masks, nurses, Canada, expedited, letter

**To the Editor:** We report results of the effect of inactivated seasonal influenza vaccination on risk of pandemic (H1N1) 2009 in a cohort of nurses in Canada who participated in a recent randomized controlled trial that compared the effectiveness of surgical masks with that of N95 respirators in preventing influenza (*1*). From September 23, 2008, through December 8, 2008, a total of 446 nurses from 8 hospitals in the province of Ontario were enrolled. They were then randomly assigned an intervention; 225 were assigned to wear surgical masks, and 221 were assigned to wear the N95 respirator. The mean age of participants was 36.2 years; 94% were women. A total of 128 (30.3%) received the trivalent influenza vaccine. Vaccination status was similar between the groups: 68 (30.2%) persons in the surgical mask group and 62 (28.1%) persons in the N95 respirator group had received the 2008–2009 trivalent inactivated influenza vaccine. The nurses were monitored from January 12, 2009, through April 23, 2009.

Blood specimens for serologic analysis were obtained before enrollment and at the end of the follow-up period. End-of-study serum samples were collected from April 23 through May 15, 2009. Serologic infection was defined by a >4-fold increase in influenza-specific hemagglutinin inhibition assay titer between baseline and convalescent-phase serum samples by using turkey **erythrocytes** and A/TN/1560/2009(H1N1), a representative pandemic influenza virus.

Of the 422 nurses included in the analysis, 42 (10.0%) showed seroconversion to pandemic (H1N1) 2009. Of 128 nurses who received the trivalent influenza vaccine, 9 (7.0%) showed seroconversion vs. 33 (11.2%) of those that did not (relative risk 0.63, 95% confidence interval 0.31–1.27, p = 0.19).

Although the point estimate was protective, the confidence interval is wide and does not exclude an increase in risk. Our sample size limits inferences that can be drawn. Heterotypic antibodies may have contributed to the relatively high rate of seroconversion. A rise in antibody titer is considered by some as an outcome associated with bias, unlike virus identification. Nevertheless, these data suggest a possible positive effect of seasonal influenza vaccine reducing risk of infection with pandemic (H1N1) 2009.
